# Cardiac radiology in Europe: status and vision by the European Society of Cardiovascular Radiology (ESCR) and the European Society of Radiology (ESR)

**DOI:** 10.1007/s00330-023-09533-z

**Published:** 2023-03-11

**Authors:** Luigi Natale, Rozemarijn Vliegenthart, Rodrigo Salgado, Jens Bremerich, Riccardo P. J. Budde, Jean-Nicholas Dacher, Marco Francone, Karl-Friedrich Kreitner, Christian Loewe, Konstantin Nikolaou, Charles Peebles, Birgitta K. Velthuis, Carlo Catalano

**Affiliations:** 1 Department of Radiological Sciences - Institute of Radiology, Catholic University of Rome, “A. Gemelli” University Hospital, Rome, Italy; 2 Department of Radiology, Medical Imaging Center, University of Groningen, University Medical Center Groningen, Hanzeplein 1, 9713 GZ Groningen, The Netherlands; 3 Department of Radiology, Antwerp University Hospital & Antwerp University, Holy Heart Lier, Belgium; 4 Department of Radiology, University of Basel Hospital, Basel, Switzerland; 5 Department of Radiology & Nuclear Medicine, Erasmus MC, Rotterdam, The Netherlands; 6 Department of Radiology, Normandie University, UNIROUEN, INSERM U1096 - Rouen University Hospital, Rouen, France; 7 Department of Biomedical Sciences, Humanitas University, Via Rita Levi Montalcini 4, 20072 Pieve Emanuele, Milan Italy; 8 Department of Diagnostic and Interventional Radiology, University Medical Center of the Johannes Gutenberg University Mainz, Mainz, Germany; 9 Division of Cardiovascular and Interventional Radiology, Department of Biomedical Imaging and Image-Guided Therapy, Medical University of Vienna, Vienna, Austria; 10 Department of Diagnostic and Interventional Radiology, University of Tuebingen, Tübingen, Germany; 11 Department of Cardiothoracic Radiology, University Hospital Southampton, Southampton, UK; 12 Department of Radiology, Utrecht University Medical Center, Utrecht, The Netherlands; 13 Department of Radiological Sciences, Policlinico Umberto I, Sapienza University of Rome, Rome, Italy

**Keywords:** Cardiovascular diseases / diagnosis, Europe, Physician’s role, Magnetic resonance imaging, Tomography, X-ray computed

## Abstract

**Abstract:**

Cardiac computed tomography (CT) and cardiac magnetic resonance imaging (MRI) are routine radiological examinations for diagnosis and prognosis of cardiac disease. The expected growth in cardiac radiology in the coming years will exceed the current scanner capacity and trained workforce. The European Society of Cardiovascular Radiology (ESCR) focuses on supporting and strengthening the role of cardiac cross-sectional imaging in Europe from a multi-modality perspective. Together with the European Society of Radiology (ESR), the ESCR has taken the initiative to describe the current status of, a vision for, and the required activities in cardiac radiology to sustain, increase and optimize the quality and availability of cardiac imaging and experienced radiologists across Europe.

**Key Points:**

*• Providing adequate availability for performing and interpreting cardiac CT and MRI is essential, especially with expanding indications.*

*• The radiologist has a central role in non-invasive cardiac imaging examinations which encompasses the entire process from selecting the best modality to answer the referring physician’s clinical question to long-term image storage.*

*• Optimal radiological education and training, knowledge of the imaging process, regular updating of diagnostic standards, and close collaboration with colleagues from other specialties are essential.*

## Introduction and purpose of this document

Cardiac computed tomography (CT) and cardiac magnetic resonance imaging (MRI) are routine radiological examinations for diagnosis and prognosis of cardiac disease, with expanding indications endorsed by multiple expert organizations [1–7]. There is an increase in the number of exams being performed (https://www.mrct-registry.org) [8–10] as well as certified cardiac imagers [11]. The expected growth in cardiac radiology in the coming years will exceed the current scanner capacity and trained workforce [12–14]. Furthermore, improvements in scanner and image reconstruction technology enable the detection of significant cardiac pathology in chest CT, which brings this prior “black box” into the diagnostic realm of any radiologist reading cross-sectional chest exams. The European Society of Cardiovascular Radiology (ESCR) focuses on supporting and strengthening the role of cardiac cross-sectional imaging in Europe from a multi-modality perspective. Together with the European Society of Radiology (ESR), the ESCR has taken the initiative to describe the status, vision and required activities in cardiac radiology to sustain, increase and optimize the quality and availability of cardiac imaging, and experienced radiologists across Europe. This document describes (1) what constitutes a cardiac CT/MRI examination, (2) the role of cardiac radiology in the radiology training curriculum, (3) the importance of coverage, image quality, and expertise in cardiac radiology, (4) a vision on collaboration in cardiac imaging with non-radiology specialties, and (5) initiatives by ESCR/ESR to support and strengthen cardiac radiology coverage and quality across Europe.

## What constitutes a cardiac CT/MRI examination?

A cardiac CT/MRI examination constitutes the entire process of determining the correct indication for the exam, exam scheduling, informing and preparing the patient, scan acquisition protocol selection, image acquisition, image reconstruction, post-processing and interpretation, and reporting and communicating the findings, and long-term storage of the report and images.

### The central role of the radiologist in a cardiac CT/MRI examination

**Fig. 1:**
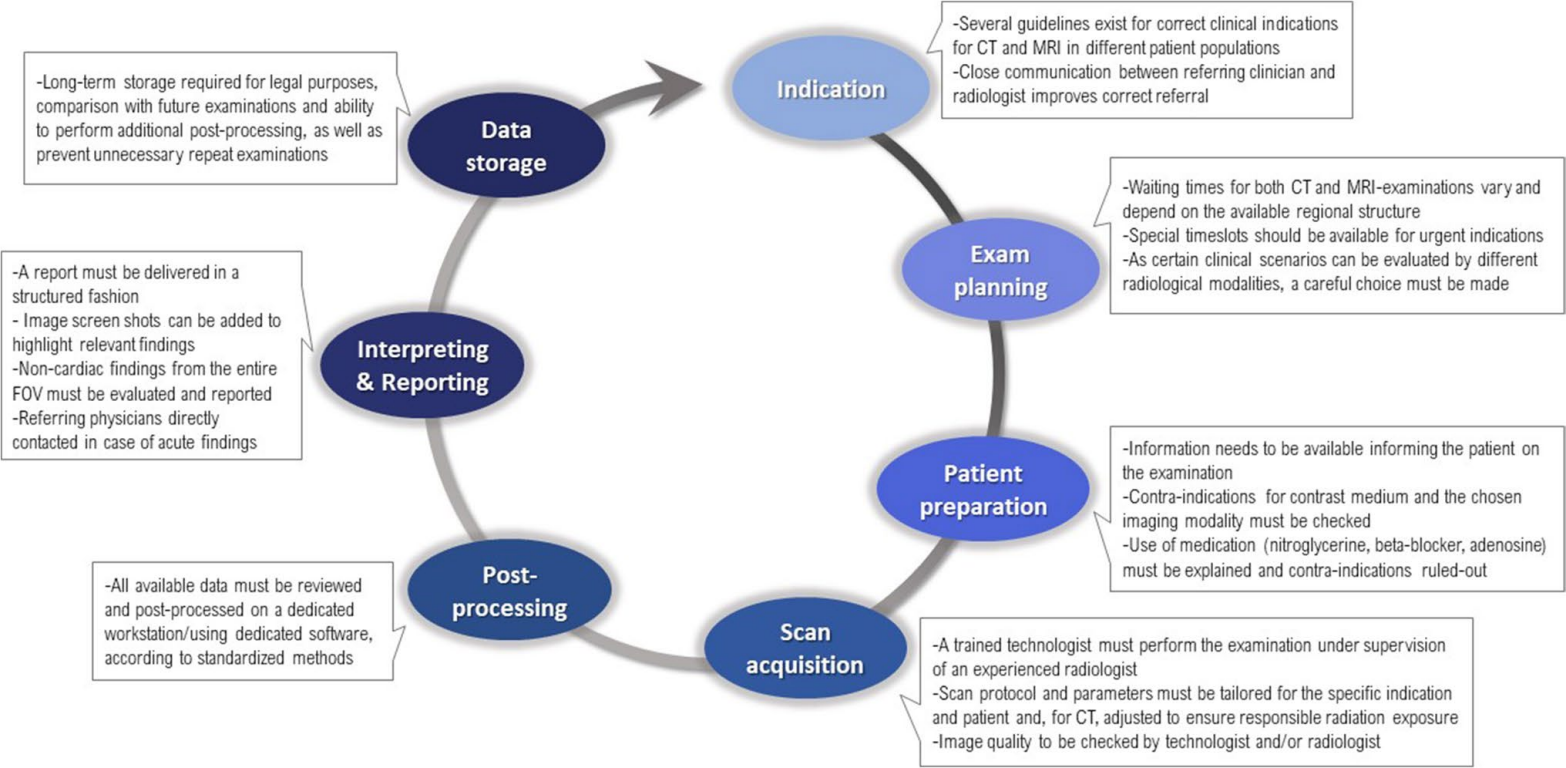
Diagnostic chain of a cardiac CT/MRI examination. CT, computed tomography; FOV, field of view; MRI, magnetic resonance imaging

Both examinations consist of a multi-stage process, starting by establishing the correct indication for each modality and ending by delivering a structured report containing all required information and long-term storage of the data. A detailed overview of all involved steps is given in Fig. 1.

The technical complexity of cardiac CT and MRI examinations, the new insights from research, and the expanding clinical indications make the radiologist a central figure in this process. The radiologist forms the link between patient, referring clinician, and performing technologist, ensuring quality control of each procedural step to deliver the best possible result for each individual patient. Making the optimal choice of modality in each patient to answer the referring physician’s question includes consideration of patient characteristics, the likelihood of adequate image quality, radiation dose, costs and scan waiting time. Knowledge of “which test to do when” is fundamental to radiology, enabling efficient use of resources without risk of self-referral, and optimal radiation protection for patients. Close interaction between technologist and radiologist helps to achieve the best image quality in each patient, which may require adjustment of routine parameters to reduce motion artefacts, etc. Interpretation of cardiac findings necessitates understanding of patient symptoms and signs, differential diagnosis, and necessary post-processing for diagnostic and prognostic purposes. Another essential step is the systematic review of extra-cardiac findings, which sometimes require additional assessment or follow-up and can also be the cause of symptoms [14]. Since the radiologist assumes a central role in diagnostic management, communication with all referring specialties is essential for patient care.

### Evolving indications for cardiac CT and MRI

Different invasive and non-invasive imaging modalities are used to evaluate cardiac anatomy and function. The applications for both cardiac CT and MRI are continuously expanding, sometimes replacing other (non)invasive modalities. In particular, CT imaging is used as a roadmap before minimally invasive intervention. An overview of routine applications is given in Tables 1 and 2. An in-depth discussion of routine and upcoming indications for cardiac CT and MRI examinations is beyond the scope of this paper.

**Table 1:** Current spectrum of indications for cardiac CT

Clinical indication	Gained knowledge
Asymptomatic individual with intermediate risk	Calcium score improves risk classification and can guide preventive therapy
Suspected coronary artery disease	Exclusion of obstructive coronary artery disease leads to more appropriate patient management Potentially obstructive coronary artery disease improves referral for angiography Detection of non-obstructive disease and high-risk plaque features offers opportunity for preventive treatment
Acute chest pain and acute coronary syndrome cannot be ruled out	Exclusion of obstructive coronary artery disease shortens length of stay in the emergency department and allows safe discharge
Recurrent chest pain after coronary intervention	Evaluation of stent and graft patency, in selected cases
Pre-procedural imaging for transcatheter valve repair / replacement	Pre-procedural planning by visualizing the target anatomy and obtaining the necessary measurements as well as evaluation of the different access routes
Prosthetic valve assessment in suspected dysfunction or endocarditis	Determination of the cause of dysfunction by visualizing the anatomic substrate and the presence of valve vegetations and perivalvular mycotic aneurysms, in selected cases
Pre-left atrial ablation	Pre-procedural planning by visualizing the interatrial septum, left atrium/appendage and pulmonary veins, and evaluating relevant anatomical variants

**Table 2:** Current spectrum of indications for cardiac MRI

Clinical indication	Gained knowledge
Ischemic cardiomyopathy	Morphological and functional evaluation, detection of post-ischemic complication, and myocardial viability assessment in a CABG candidate
Non-ischemic cardiomyopathy	Morphological and functional assessment, detection of a possible underlying pathological substrate and potential classification of the involved cardiomyopathy, as well as prognostic factors for events
Myocardial ischemia	Detection of hemodynamic significance of coronary artery disease
Heart failure	Morphological and functional assessment, detection of possible underlying pathological substrate and thrombus, and evaluation of potential etiology
Valvular heart disease	Visualization and quantification of transvalvular flow, and evaluation of functional repercussion on and remodeling of the cardiac chambers
Congenital heart disease	Knowledge about cardiac anatomy and suspected defects. Pre-operative guidance (including shunt fraction determination in atrial and septal defects) and long-term follow-up into adulthood

## Role of cardiac radiology in training curriculum

Facing the expected growth in cardiac radiology, it is important to strengthen cardiac imaging as one of the key areas of radiology in the training curriculum of national radiological societies. During the board certification process for radiology, all trainees should become proficient to supervise and report cardiac CT and MRI examinations. It is essential to teach the whole diagnostic chain (Fig. 1) in residency.

The ESR’s updated European Training Curriculum (ETC) (https://www.myesr.org/education/training-curricula) shows how to integrate cardiac imaging into the radiology curriculum. It defines three levels, whereby levels I and II should be part of the board certification process. In level I (years 1–3), the trainee develops core knowledge of the relative value of imaging modalities for the heart and great vessels. This includes an understanding of basic cardiac physiology, anatomy, and common normal variants. This knowledge forms the basis for further training and provides transferable skills. In level II (years 4 and 5), the trainee acquires in-depth knowledge of the pathophysiology and management of chronic coronary syndromes, valvular heart diseases, cardiomyopathies, and congenital heart diseases. Furthermore, residents learn to supervise more advanced cardiac imaging techniques including stress cardiac MRI, and apply pharmacological strategies like adenosine infusion.

Level III training is a full-immersion training in the subspecialty of cardiovascular radiology. This training is competency-based and may be performed in a modular fashion where subspecialty competencies can be obtained over a period of time, partly within level II, with unrestricted extra training of at least 1 year after board certification. A logbook of activity should be maintained during the training period, providing a formal validated record of competencies achieved, examinations performed, and a basis for regular training assessment. A final subspecialty examination can only be done after all competency requirements are fulfilled.

In 2011, the ESCR introduced the European Board of Cardiac (later renamed Cardiovascular) Radiology (EBCR) Diploma, a recognized European qualification that is endorsed by the ESR. Importantly, as cardiac CT and MRI are part of routine radiology training (like CT pulmonary angiography), radiologists do not need specific certification to perform these examinations. However, for subspecialized colleagues such as level III trained fellows, the EBCR diploma provides special recognition of advanced skills and competencies in cardiovascular radiology.

Cardiac training during the radiology residency is quite diverse in European countries. A recent survey conducted by the ESCR and ESR regarding cardiac radiology training in European member countries (unpublished data) indicates that most European countries provide level I training (both cardiac CT and MRI), and 40–45% also provide level II training. However, a dedicated training curriculum in cardiac radiology is not provided in all countries in Europe. The ETC is well suited to bring countries together for the implementation of cardiac imaging as an important part of general radiology.

(Continuing) Education on novel, proven techniques in cardiac CT and MRI is essential for radiologists in training and practicing radiologists, particularly in view of fast developments in hardware and software possibilities, and guideline updates. Both the annual ECR congress and ESCR meeting (see below) provide an optimal setting to obtain this regular update, including workshops for hands-on experience in the newest techniques.

## Importance of coverage, image quality, and expertise

### Coverage

Providing adequate availability for performing and interpreting cardiac CT and MRI is essential, especially with expanding indications. For instance, the routine use of CT for coronary assessment is expected to increase substantially. Providing a cardiac imaging service includes everything from managing the equipment, technicians, and physicians as well as picture archiving and retrieval; this fits perfectly in the existing infrastructure of radiology departments. Most current CT and MRI scanners are technically sufficient. However, due to the intensive use of scanners for other body areas, sufficient slots need to be allotted for cardiac examinations to reduce waiting times as a bottleneck for cardiac imaging. Another important aspect is having sufficient technologists and radiologists with cardiac CT/MRI experience in each hospital. Besides creating a prominent role for cardiac imaging in the radiology training curriculum, the training of technologists should also become a focus.

### Image quality and reporting quality

Cardiac imaging is more technically challenging than most other radiological studies due to the continuous cardiac and respiratory motion. Image acquisition needs to be synchronized to the electrocardiogram (ECG) signal and/or respiratory motion. A good knowledge of CT and MRI techniques and expertise in optimizing scan acquisition is required. An optimal balance between image quality and radiation exposure is necessary for cardiac CT. The scan acquisition protocols may need to be tailored to the individual patient (e.g. performing coronary CT angiography in systole in patients with high heart rates), with close collaboration between technologist and radiologist and available supervision at the scanner.

Good image quality is also essential for image post-processing as quantification may directly influence patient management (e.g. cardiac function in MRI). Post-processing requires standardized execution with a chosen software package to make sure consistent and accurate measures are obtained. Post-processing results should be saved to allow a comparison for future examinations.

ESCR strongly recommends a structured approach to reporting findings. This ensures that all relevant items will be covered in the report, guides less-experienced radiologists in making a complete evaluation of the examination, and provides a consistent and reproducible format to the referring clinical specialist for review. ESCR provides its members with electronic templates in a joint venture with Smart Radiology [17]. Apart from structured reporting, ESCR encourages classifications such as the Coronary Artery Disease – Reporting and Data System to facilitate communication and standardization [15]. Advances in the automation of analyses (including CT plaque assessment and MRI function assessment) will allow a broader range of users (such as trained radiographers) to contribute to structured reports.

### Expertise

Next to increasing dedicated cardiac imaging examinations, newer and faster scanners often depict the heart in detail on scans performed for other reasons (e.g. oncological patients). The heart should be carefully examined, especially as symptoms for which patients undergo chest imaging may be explained by cardiac findings (e.g. myocardial infarction on CT pulmonary angiography). This means that any radiologist involved in reporting chest examinations should be able to evaluate the heart. Vice versa, unexpected findings outside the heart are frequently encountered on cardiac imaging and require adequate assessment, as they may need follow-up (e.g. lung or liver lesions) or may be the explanation for a patient’s symptoms (https://www.myesr.org/education/training-curricula). Radiologists possess the required knowledge to interpret the examination as a whole and relate findings to prior imaging in the picture archiving and communication system (PACS). This may prevent the need for lengthy and costly follow-up or additional analysis. Furthermore, most (cardiac) radiologists are CT and MRI experts beyond the heart, understand the complexity of new techniques such as spectral CT and MRI mapping, and have ample knowledge of monitoring radiation dose and contrast material exposure.

Artificial intelligence (AI) techniques will become increasingly embedded in non-invasive imaging as a whole and require special expertise. The radiologist will likely become more of a process manager and eventually, second rather than first reader of the scan. Incorporating AI techniques—often not restricted to one body area—will also require an infrastructure integrated within PACS that is thus best placed within the radiology department.

## Vision on collaboration with other specialties

### Collaboration with related specialties

Cardiac radiologists must combine their knowledge and skills in imaging modalities to bring relevance to their reports and input to referring physicians and multidisciplinary meetings. Besides radiologists, many medical professionals are involved in cardiac imaging: nuclear medicine physicians, (interventional) cardiologists, pediatric cardiologists, vascular internal medicine physicians, and cardiac/vascular surgeons, with variations among European countries. All these specialists rely on high-quality imaging, and in some cases perform and report the studies. These dual roles require detailed clinical and imaging knowledge, including awareness of guidelines and appropriateness criteria. For these reasons, close collaboration among specialists is crucial, while maintaining full respect for each other’s expertise area.

Imaging and clinical fundamentals necessary for cardiac imagers include.

- good knowledge of cardiac anatomy, common variants, and cardiac physiology;
- common terminology in order to speak the same language: cardiac imaging planes, myocardial segmentation, and the acquired functional parameters have to be harmonized, with known reference values, for all imaging modalities;
- knowledge of the correct indications of each imaging modality, including strengths and weaknesses of each modality, as well as the role of each imaging technique in different patient pathways;
- knowledge of current trials and guidelines related to the area of expertise, as well as in the field of related imaging modalities; and
- screening for relevant extra-cardiac findings in all scans.

### Heart team involvement

Aligned with the development of multidisciplinary tumor boards, the heart team has become a clinical necessity in cardiology and cardiovascular surgery. In the era of individually tailored treatment plans for patients, the decision to commence many invasive and non-invasive therapies will benefit from a multidisciplinary team discussion. This team approach is already utilized by many centers of excellence, where optimal results require not only highly experienced clinicians but also dedicated cardiac imagers. The contribution of each specialist, with specific knowledge and competence, becomes crucial for the choice of management, interventional procedures, and surgery planning. Cardiac imagers add value by discussing complex multi-modality imaging results, imaging results for pre-procedural planning, interventional guidance, and advice on post-procedural follow-up, e.g. in ablation procedures and structural heart disease treatment. The involvement of a dedicated cardiac radiologist within the multidisciplinary team brings many advantages including, multimodality knowledge, expertise in 2D and 3D image interpretation, detailed knowledge of radiation exposure/-control, and expertise in the interpretation of non-cardiac findings. On the other hand, in many hospitals, the workload for radiologists currently limits participation in more multidisciplinary meetings. This means there must be a shift in radiology towards seeing participation in multidisciplinary meetings as important as the reporting workload, coupled with adequate reimbursement.

If the goal is a full integration of radiologists in the heart team, part of the educational and training programs should be inter-disciplinary, with different specialists involved both in teaching and in learning, where for example the role and findings on echocardiography are taught by a cardiologist, together with teaching on radiological cardiac imaging by a radiologist. The increasing role of CT and MR gives a unique opportunity to radiological societies to take the lead in this process.

## ESCR/ESR activities to support and improve coverage and quality across Europe

ESCR is the reference medical society of cardiovascular radiology in Europe, dedicated to supporting science, teaching, research, and quality of service in the field. This mission requires the development of activities to strengthen the international outreach of society and to support the clinical value of advanced cardiac Radiology in healthcare.

### Training and education

Expanding education and training opportunities with the clear vision of standardized education of radiologists in Europe is the first aim to achieve quality improvement and high standards of clinical service in cardiac imaging.

ESCR offers cardiac imaging scholarships and fellowships, in collaboration with the European School of Radiology (ESOR). These are 3-month opportunities available for selected candidates within the first 3–4 years after board certification. Training is offered in expert training centers in Europe, under the supervision of qualified tutors. Programs are structured into intensive weekly modules, combining image analysis and post-processing with assisted reporting. At the end of the training period, the trainee will have competence in discussing the appropriate imaging modality and imaging technique with referring clinicians and have the expertise to interpret diverse cardiac examinations.

Onsite initiatives also include the annual meetings that have been organized by ESCR since the first edition in Berlin in 2002. These congresses constitute an important annual event for cardiac radiologists in Europe with ample networking opportunities. To promote the concept of a cardio-thoracic imaging specialist and to strategically intensify the cooperation with a sister subsociety, ESCR organizes periodical joint meetings with the European Society of Thoracic Imaging (ESTI), the next to be held in 2023.

ESCR has offered monthly online webinars since 2015, guided by educational and scientific committees. The webinars are oriented towards different levels of expertise (basic and advanced) and offer a wide array of topics that are becoming more multidisciplinary, with the contribution of internationally renowned speakers. During the pandemic years, online training became a necessary and important resource to continue medical education; webinars were combined with fully virtual hands-on workshops and meetings, which were offered as a combination of live and recorded sessions. The development of live case discussion sessions has been a more recent achievement, aiming to increase the interactivity of our participants and to offer a more “real-lif-based” training experience. ESCR believes that online learning is a flexible and effective source of teaching that increases the international outreach of society and favors accessibility to initiatives along with less use of resources and time.

ESCR and ESR are convinced that training non-cardiac radiologists will help support cardiac imaging practice and availability across Europe. Accordingly, workshops and courses for non-cardiac radiologists are offered, either during the annual meetings as pre-conference sessions or online workshops, e.g. during the European Congress of Radiology 2021 and 2022 winter editions.

### Networking including the Young Club

In response to growing interest and increased participation of younger members, ESCR has formed a Young Club, which is structured as a working group of medical or PhD students, residents, and young radiologists until the age of 35 years. The Young club aims to create connections and foster collaborations between young specialists and researchers, by providing an international communication platform to share experiences, exchange ideas and knowledge, and opportunities for social interaction. Members can interact with key opinion leaders through dedicated mentorship programs to manage a digital bulletin to be distributed to all members with the monthly ESCR newsletter and to participate in ESCR activities including the organization of webinars and dedicated sessions at the annual conference.

### Consensus documents on imaging protocols and reporting, and involvement in clinical consensus documents involving radiological diagnostics

Scientific research and consensus document preparation are keys to raising ESCR’s international authoritativeness, reinforcing cooperation with cardiac imaging societies, and providing clinical guidance in cardiac imaging. This fundamental and crucial step helps to standardize technique and reporting among European radiologists. In recent years, ESCR has contributed to many publications including consensus documents and scientific articles based on the ESCR registry, published in major radiology and cardiology journals [5, 9, 16–24]. These documents include topics ranging from quality and safety in cardiac CT and MRI [10, 16, 17], standardization of imaging methods and reporting [5, 23, 24], guidance on imaging during the COVID-19 pandemic [19], and position documents on innovations: machine learning and cardiac PET-MRI imaging [18, 21]. As the sole multimodality-based cardiovascular radiology society in Europe, we have expanded towards extra-cardiac areas with the publication of consensus documents on carotid imaging [23, 24]. Other vasculatures like the aorta and peripheral vessels are routinely assessed with advanced imaging studies and are part of the cardiovascular radiology practice. Clinical practice is routinely adapted to imaging indications reported in cardiac/cardiovascular guidelines, making CT and MRI key tools in the diagnostic algorithm of most clinical scenarios. For this, new appropriateness criteria regarding cardiovascular CT and MRI practice will be developed.

## Conclusion

The ESCR and ESR strongly support the increasing clinical role of non-invasive cardiac CT and cardiac MRI. The radiologist is ideally situated to objectively assess the best advanced non-invasive imaging modality to answer the referring physician’s clinical question. This role can only be guaranteed by an optimal education and training process during and after the radiology specialization, deep knowledge of the imaging process, regular update of diagnostic standards, and close collaboration with colleagues from other specialties, with respect for each other’s clinical expertise and role. This position statement summarizes instruments to achieve these goals, from training programs to other educational activities, standardization, and consensus documents.

## Abbreviations

AI: Artificial intelligence
CT: Computed tomography
EBCR: European Board of Cardiovascular Radiology
ECG: Electrocardiogram
ESCR: European Society of Cardiovascular Radiology
ESOR: European School of Radiology
ESR: European Society of Radiology
ESTI: European Society of Thoracic Imaging
ETC: European Training Curriculum
FOV: Field of view
MRI: Magnetic resonance imaging
PACS: Picture Archiving and Communication System

## Declarations

### Guarantor

The scientific guarantor of this publication is R. Vliegenthart.

### Statistics and biometry

No complex statistical methods were necessary for this paper.

### Methodology

• Not applicable
